# Impaired Tilt Perception in Parkinson’s Disease: A Central Vestibular Integration Failure

**DOI:** 10.1371/journal.pone.0124253

**Published:** 2015-04-15

**Authors:** Giovanni Bertolini, Andrea Wicki, Christian R. Baumann, Dominik Straumann, Antonella Palla

**Affiliations:** Department of Neurology, Zurich University Hospital, Zurich, Switzerland; Oslo University Hospital, NORWAY

## Abstract

**Introduction:**

Impaired balance control is a hallmark symptom in Parkinson’s disease (PD). Altered sensory-motor integration contributes to the deficiency. We aimed to determine whether impaired vestibular signal processing added to the disorder. We exposed patients (N = 11; 68±6y) and age-matched healthy subjects (hS: N = 19; 65±11y) on a motion platform in complete darkness to two consecutive forward tilt movements (12 series; N = 24; overall 288 trials) and asked them to indicate which tilt was perceived larger. By combing tilt movements with translations we manipulated vestibular sensory input in order to investigate whether putative impairment resulted from a deficiency of the sensory organs (semicircular canals in ‘single-SCC-cue-condition’, otoliths in ‘single-OT-cue-condition’) themselves or to a sensory integration failure (‘multi-cue-condition’).

**Results:**

Tilt discrimination in the multi-cue-condition was inferior in patients compared to hS (p = 0.02). No significant differences between the two groups were found for both single-cue-conditions. Comparison of multi-cue-condition with a prediction resulting from the combination of both single-cue-conditions by optimal observer theory revealed that patients (p = 0.04), in contrast to hS, failed to efficiently combine SCC and OT information to improve tilt perception.

**Conclusion:**

We found that PD patients distinguished forward tilts less precise than hS, suggesting impaired vestibular perception. Tilt discrimination in patients, moreover, did not improve as much as in hS in conditions where both SCC and OT information was available compared to conditions where only SCC or OT cues were activated. The latter provides evidence that tilt misperception in PD most likely results from an integration failure of vestibular signals.

## Introduction

Postural instability is a hallmark symptom in patients affected by Parkinson’s disease (PD). The underlying etiology is complex, involving various interacting neuronal systems such as the motor and sensory systems, subcortical regulatory mechanisms, and also higher cortical functions implicated in mental processes for adaptation to environmental changes (see Boonstra et al. 2008 for a recent review [1]). While postural instability was once thought to be caused by primary motor system impairment, there is now broad agreement that it consists of an integration failure of sensory-motor coordination in subcortical regulatory mechanisms (see Benatru et al. 2008 and Boonstra et al. 2008 for recent reviews [1,2]). Postural control is a complex neural process based on the accurate interpretation of convergent sensory information originating from vestibular, visual and proprioceptive sensors and the generation of adequate motor responses. Each sensory signal alone, however, provides ambiguous information. The visual system, for example, cannot differentiate between movements of the visual world and self-motion since both lead to retinal displacement of visual objects. Only the additional information from other sensory modalities (e.g. vestibular and proprioception) resolves this uncertainty and allows the generation of the appropriate motor response for balance control.

Experiments manipulating sensory information demonstrate deficiencies of PD patients in accurately integrating incoming sensory signals. When exposed to displacements of the visual environment while standing on a firm platform, PD patients, for example, show abnormally increased body sway suggesting an abnormal dominance of visual information for postural control [3]. When asked to maintain upright stance during slow platform rotatory oscillations along the lateral and antero-posterior planes in complete darkness, i.e. by relying mainly on proprioceptive sensory inputs, PD patients also perform worse than healthy controls [4]. PD patients, thus, seem unable to accurately integrate and maybe even more importantly to prioritize visual and proprioceptive information when one of both becomes unreliable.

The impact of vestibular information within this integrative process is less clear. To characterize the vestibulo-spinal contribution to balance control in patients with PD, Pastor et al., for example, investigated postural sway after galvanic stimulation of the vestibular nerve [5]. No differences were found between PD patients and healthy controls. Based on this result, the authors concluded that vestibular dysfunction does not account for PD-associated postural deficits (see also [6,7] for other examples of normal vestibular function in PD). In contrast, Pollak et al. in a more recent study found abnormal vestibulo-collic responses that were assessed via vestibular evoked myogenic potentials suggesting that postural imbalance in PD patients could arise, at least partially, from vestibulo-spinal pathway impairment [8] (see also [9,10] for other examples of vestibular dysfunction in PD). One possible explanation for the persistent contradictory findings could stem from the quasi-static experimental setup used in most studies. Vestibular function, in fact, becomes more important during dynamic situations as can be seen in vestibular-loss subjects being stable on a flat firm surface and unstable whenever the surface moves [11,12]. Deficiencies of the integrative process of the vestibular system in PD patients, if present, would presumably be more likely unmasked during dynamic stimulations. Evidence pointing towards this was recently provided by De Nunzio et al. [13]. The authors observed that PD patients oscillated more than polyneuropathic patients when standing on a moving platform. They reasoned that polyneuropathic patients, in the absence of proper proprioceptive inflow, had increased their ability to use vestibular cues for dynamic postural control, while PD patients, despite proper peripheral proprioceptive and vestibular sensory input, failed in doing so.

In this study, we aimed to investigate the contribution of vestibular signals in the sensory integration process in patients affected by PD. We hypothesized that patients fail to appropriately integrate vestibular signals during dynamic perturbations and predicted impaired performance when asked to estimate pitch tilts that predominantly probe the vestibular system.

## Material and Methods

### Standard protocol approvals and patient consents

Written informed consent was obtained from all participants after full explanation of the experimental procedure. The protocol was approved by the Ethics Committee of the Canton of Zurich, Switzerland, and was in accordance with the ethical standards of the 1964 Declaration of Helsinki for research involving human subjects.

### Study cohort

Eleven patients affected by idiopathic Parkinson’s disease (two females; mean age ± SD: 68 ± 6 years) and 19 age-matched healthy subjects (9 females; mean age 65 ± 11 years) participated in the study. Diagnoses were made in accordance with clinical criteria defined by the UK PDS Brain Bank.

All patients received a standard clinical examination, including detailed history taking and investigation for clinical signs of vestibular, cognitive (by the Mini Mental Status Exam) and motor deficits (by Unified Parkinson Disease Rating Scale part 3 motor section). Patients average disease duration was 6.8 years (SD = 3.8, range 2 to 15), Mini Mental Status (MMS) Exams ranged from 26–30 (mean ± SD: 28.1 ± 1.4; normal cut-off set to 25 according to Dubois et al. [14]; in patient ID3 the MMS score was not assessed, but during clinical examination the patient appeared to have normal cognition), and Unified Parkinson Disease Rating Scales from 12–39 (mean ±SD: 22 ± 9.2). No patient had clinical signs of central and / or peripheral nervous system impairment other than the ones expected in Parkinson’s disease (PD), in particular, all showed normal pallesthesia, vestibular function as well as muscle strength and sensation. All patients were left on regular medication and tested in their ON-condition. Detailed patients characteristics are given in S1 Table of supplemental data.

### Experimental setup

Subjects were seated on a chair mounted on a six-degree-of-freedom motion platform (E-Cue 624–1800 motion system, built by FCS Simulator Systems, Schiphol, Netherlands). A four-point safety harness secured the subjects. Their head was additionally immobilized by an individually molded thermoplastic mask (Sinmed, Netherlands). White noise was transmitted via earphones to mask acoustic cues from the electromechanical actuators. All experiments were conducted in complete darkness. One to four practice sessions were given to each participant before data collection and no feedback about the subjects performance was given.

### Experimental protocol

Pitch tilts lead to changes of the head relative to gravity activating both the semicircular canals (SCC; activated by angular acceleration) and the otolith organs (OT; activated by changes of gravitational force). Sensing pitch tilts requires an accurate central integration of both sensory organs as both sensory cues by themselves are insufficient to accurately detect tilt movements. Specifically, SCC, functioning like gyroscopes, provide only transient tilt velocity signals while OT, as any accelerometer, cannot distinguish between tilts of the gravito-inertial vector (vector sum of gravity and inertial accelerations) due to changes of orientation with respect to gravity or due to linear accelerations (Einstein equivalence principle [15]).

**Fig 1:**
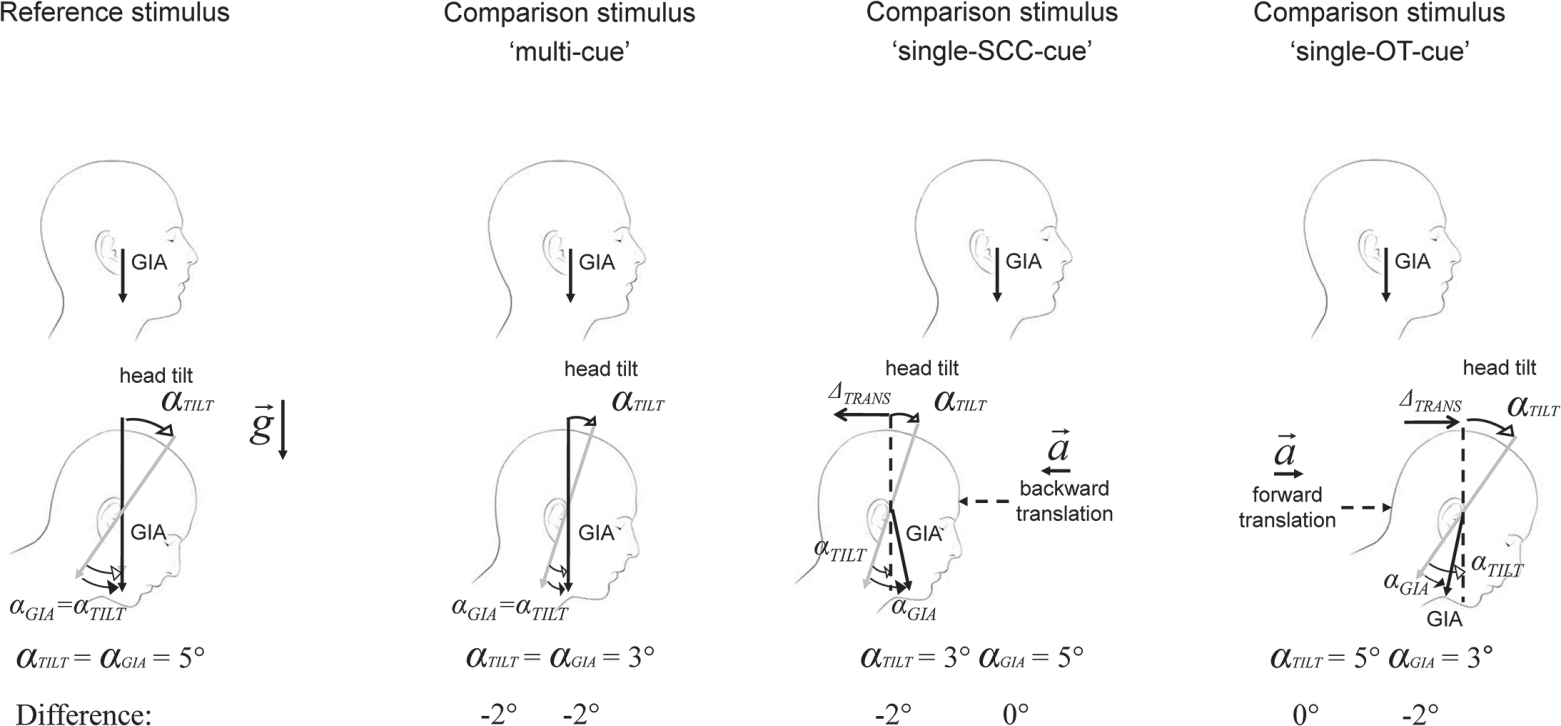
Schematic illustration of tilt movements in the reference and comparison conditions with corresponding vestibular organ activation. Upper row: initial head position; lower row: final head position. GIA = gravito-inertial acceleration; αTILT: head rotation angle in degrees; αGIA: rotation angle of the GIA vector. Comparison stimulus ‘multi-cue’: ‘Pure’ forward tilt movement. The tilt movement activates both SCC and OT as in the reference stimulus. Differences between both conditions result from the tilt angle, which, in this example, is smaller than the reference tilt angle (i.e. 3° vs. 5° tilt). Comparison stimulus ‘single-SCC-cue’: Combined tilt-translational movement. The backward translational movement modifies the αGIA. Movement parameters are thereby chosen to provide the same OT activation as in the reference stimulus (i.e. αGIA = 5°). Perceptual differences between this and the reference tilt stimulus thus result from SCC activity (i.e. αTILT = 3°). Comparison stimulus ‘single-OT-cue’: Combined tilt-translational movement. The forward translational movement modifies the αGIA providing different OT activation (i.e. αGIA = 3°). SCC activation (i.e. αGIA = 5°) is instead equal to the reference stimulus as the tilt angles are equal in both conditions. Difference: values represent the difference of vestibular, i.e. SCC and OT, activation between the reference and the stimulus.

Computational models, supported by neurophysiological data, have shown that this motion ambiguity can be solved by central integration, i.e. by combining SCC and OT signals (see Angelaki and Yakusheva 2009 for a recent review on that topic [16]). Following our hypothesis of an impaired integrative capacity of the vestibular system we thus additionally exposed patients to pitch tilts consisting of combined tilt—translational movements to either cancel OT or SCC information (see Fig 1 for explanation). These stimuli allowed investigating whether the predicted misperception was most closely the resultant of an integration failure or whether it was due to a failure of the peripheral vestibular system.

Repetitive trials of two consecutive forward pitch tilts, i.e. forward head rotations about the earth-horizontal inter-aural axis, were provided to each subject. Subjects were asked to indicate which of the two consecutive motion stimuli provided the larger forward pitch tilt (two-alternative forced-choice task- 2AFC) [17]. Answers had to be given within three seconds after the second forward pitch tilt. Subjects thereby pressed the right button of a two button panel mounted in front of them, if the first movement was felt larger, and the left button, if the second movement was felt larger.

Motion stimuli: In each trial one of the two motion stimuli was always a reference stimulus (described below) while the other was one of twelve comparison stimuli. The twelve comparison stimuli were grouped in three conditions according to the underlying vestibular activation pattern; these conditions were termed: ‘multi-cue’, ‘single-SCC-cue’ and ‘single-OT-cue’ (see below for details on the kinematics of the motion stimuli and Fig 1 for a schematic illustration). The order of presentation of the reference and comparison stimuli varied pseudo-randomly from trial to trial. Likewise, the trials of each condition were pseudo-randomly intermingled. Each trial was repeated 24 times. Overall 288 trials were given with no performance feedback. The duration of the motion stimuli was approximately 1 second (the maximal difference of time between motion stimuli was 22 miliseconds).

Reference stimulus: ‘pure’ 5 degree forward tilt along the sagittal plane (velocity trapezoid with peak acceleration 90 deg/s^2^; peak velocity 5 deg/s). During this stimulus semicircular canal (SCC) activation, due to the pitch angular velocity, and otolith (OT) activation, due to the tilt with respect to gravity, code the same tilt angle (i.e. 5 deg).

Comparison stimulus ‘multi-cue’: ‘pure’ forward tilt movement with amplitudes of 3, 4, 6 or 7 degrees (velocity trapezoids with peak acceleration 90 deg/s^2^; peak velocity 3, 4, 6 or 7 deg/s—see Fig 1). Note that, as motion profiles differed by tilt angle and peak velocity but had the same peak acceleration, this resulted in small differences of motion duration between the comparison and reference stimuli (difference: 11 milliseconds for a 4 and 6 deg comparison to 5 deg reference stimulus; 22 milliseconds for a 3 and 7 deg comparison to 5 deg reference stimulus). As for the reference stimulus, this stimulus activated both OT and SCC.

Comparison stimulus ‘single-SCC-cue’: movement combination of ‘pure’ tilt and forward or backward translation (total displacement: ± 3 or ± 6 cm) in order to modify the direction of the resultant gravito-inertial acceleration by ± 1 or ± 2 degree, respectively. Tilt amplitudes (and relative peak velocities) in this condition were as in condition ‘multi-cue’ (i.e. 3, 4, 6 or 7 degree); the additional translational movement (total displacement: ± 3 or ± 6 cm; peak velocity ±0.08 or ±0.17 m/s; peak acceleration ±0.17 or ±0.34 m/s^2^) modified the linear acceleration so that the ‘single-SCC-cue’ stimulus provided the same OT activation as in the reference stimulus (or in other words, the gravito-inertial acceleration tilted with the exactly the same motion profile as during the reference stimulus; see α GIA in Fig 1 for Reference stimulus and for Comparison stimulus ‘single-SCC-cue’). As for the ‘multi-cue’ condition differences of motion duration between the comparison and reference stimuli were 11 and 22 milliseconds. To indicate whether the comparison stimulus was larger or smaller than the reference stimulus, therefore, in ‘single-SCC-cue’ subjects could only rely on SCC information (since OT information was equal in the reference and comparison ‘single-SCC-cue’ condition).

Comparison stimulus ‘single-OT-cue’: movement combination of ‘pure’ tilt and forward or backward translation (total displacement: ± 3 or ± 6 cm). Tilt amplitude in this condition was as in the reference stimulus (i.e. 5 deg; velocity trapezoid with peak acceleration 90 deg/s^2^; peak velocity 5 deg/s); the translational movement (total displacement: ± 3 or ± 6 cm; peak velocity ±0.08 or ±0.17 m/s; peak acceleration ±0.17 or ±0.34 m/s^2;^ as for the other two conditions differences of motion duration between the comparison and reference stimuli were 11 and 22 milliseconds) instead modified the ‘single-OT-cue’ in order to provide different OT activation as in the reference stimulus. Thus, to indicate the larger or smaller tilt subjects had to rely only on OT information.

### Data analysis

**Fig 2:**
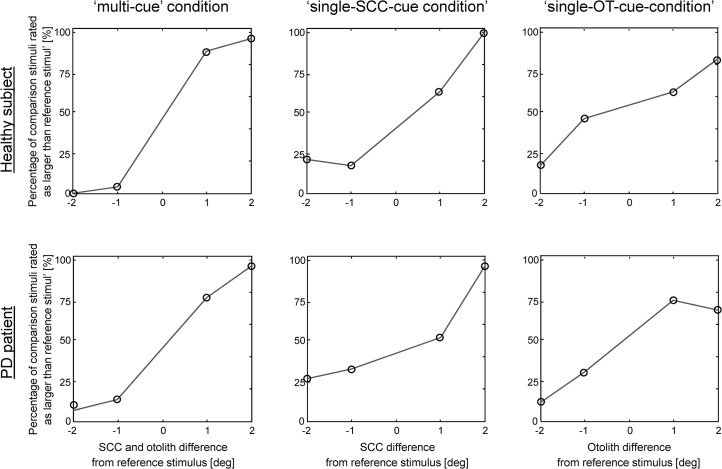
Discrete approximation of the psychometric curves (dots connected by gray lines) in one healthy subject (upper panel) and one PD patient (patient index ID5; lower panel) plotted for the three conditions in separate three separate graphs (from left to right: ‘multi-cue’, ‘single-SCC’ and single-OT condition. Abscissa: difference of SCC and/or OT activation between the comparison and reference stimulus. Ordinate: percentage of times the comparison stimulus was rated as larger tilt movement than the reference stimulus. Each of the four data points in each graph correspond to percentage recorded in all the trials of one the four comparison stimuli tested in that condition.

**Fig 3:**
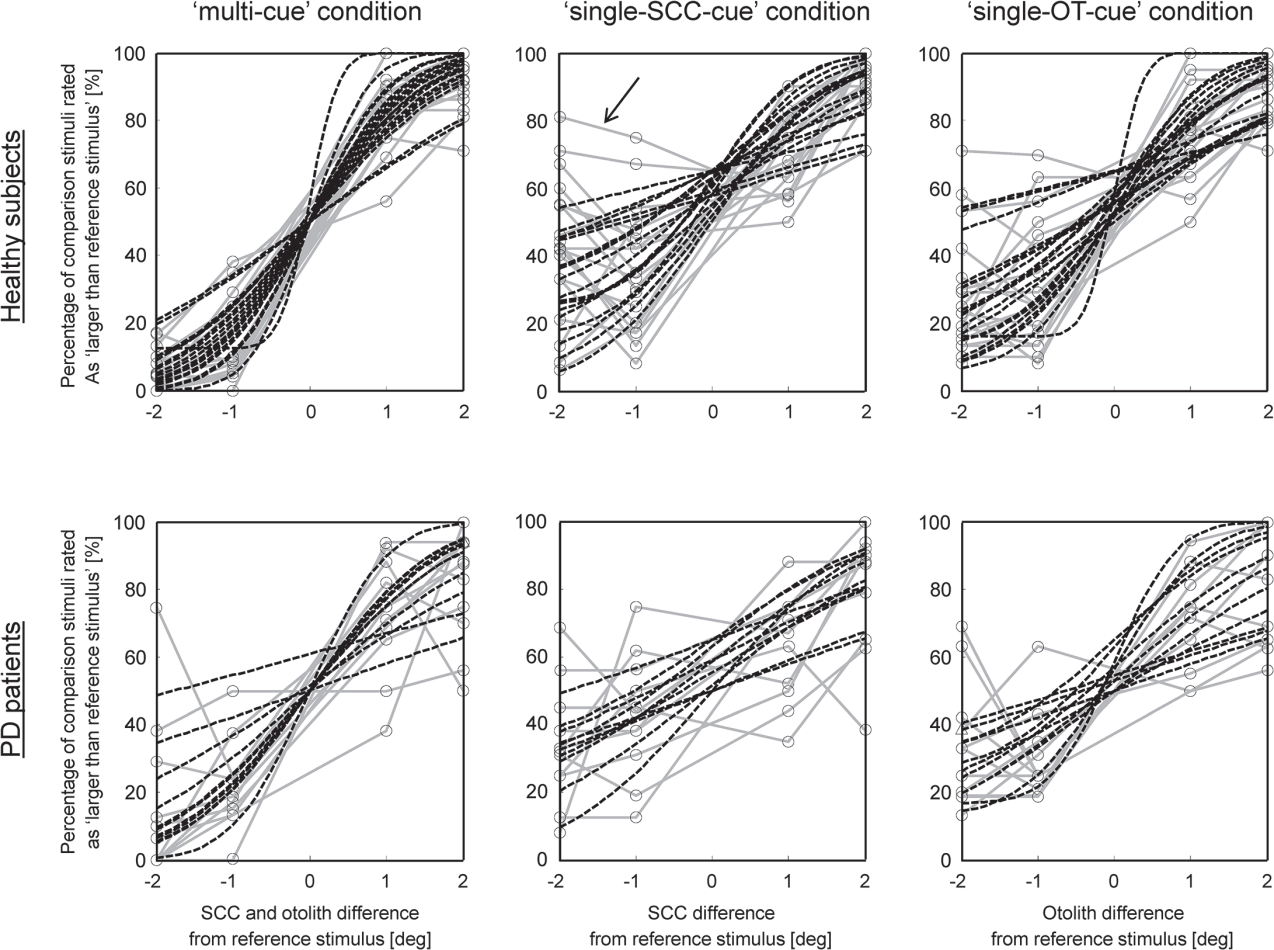
Discrete approximation of the psychometric curves (dots connected by gray lines) and cumulative Gaussian fit of the data representing the psychometric curves (dashed curves) in all healthy subjects (upper panel) and PD patients (lower panel). Abscissa and ordinate as in Fig 2. Arrow: indicates the increased uncertainty during the backward translational—tilt movement compared to the forward translational—tilt movement, although the difference of vestibular activation between the comparison and reference stimulus was equal (see text for further explanation).

The number of times subjects rated the comparison stimulus larger than the reference stimulus for each stimulus condition within each of the three comparison motion profiles was calculated to quantify the behavioral performance of tilt perception. The healthy subject in Fig 2, for example, never rated the comparison stimulus larger than the reference stimulus in the ‘multi-cue’ condition in the trials with a 3 deg (i.a. smaller) comparison and a 5 deg (i.e. larger) reference stimulus. Trials were presented 24 times and the overall performance was expressed as a proportion (parameter *y* in Eq 1 below) by dividing the number of errors (zero for the healthy subject) by the number of times the trial was presented (24). Note that the performance of the healthy subject was imperfect for the ‘multi-cue’ condition trials of 7 deg comparison vs. 5 deg reference stimulus. Specifically, the subject only rated 23 from 24 trials correctly. We fitted a psychometric curve (see Fig 3) to the data, and the slope of the psychometric function provides a robust measure of the subjects performance, combining the error information from all comparison-reference trials in a unique parameter (see Ref. [18] for an extended review about the advantages and disadvantages of psychometric fitting over raw data analysis).

The data of each condition were fitted with a cumulative Gaussian curve [18] (Eq 1) for each subject, with the vestibular signal difference between comparison and reference stimulus (i.e. ± 1 or ± 2 degree; see also values of ‘Difference’ in Fig 1) as independent variable.

$$y=\lambda +\frac{1-\lambda }{2}\left(1+erf\left(\frac{x}{\sigma \sqrt{2}}\right)\right)$$ Eq. 1

*x* is the vestibular signal difference, *erf* represents the error function, λ the proportion of lapses, i.e. the stimulus independent errors, and σ determines the slope the psychometric function. A maximum likelihood estimation was used to obtain estimates of σ and λ for each subject in each condition, assuming that answering was a Bernoulli process with a probability p(x) that the subject indicates the comparison stimulus as bigger [18].

#### Comparison of ‘multi-cue’ condition with ‘single-cue’ conditions by optimal observer theory prediction

The parameter σ, determining the slope of the cumulative Gaussian curve also represents the standard deviation of the Gaussian distribution. This latter reflects, in our experiment, the inherent degree of uncertainty in the subject’s response, or in other words, the subject’s estimate of comparison vs. reference stimulus based on its sensory information. Thus, the higher σ, the flatter the slope, and, hence, the major uncertainty of tilt perception (see also Fig 3). Since σ in the ‘single-SCC-cue’ and ‘single-OT-cue’ condition refers to the uncertainty in the estimate based on ‘only’ SCC or OT activation, σ will be referred in the following as σ_SCC_ and σ_OT_, respectively. In the ‘multi-cue’ condition both, SCC and OT contribute to the estimate; σ will therefore be termed σ_SCC+OT_.

Optimal observer theory can be used to investigate whether the combination of independent sources of information reduce the uncertainty of response that is inherent within these sources. Originally used in sound and visual perception it has been successfully applied in vestibular research (see Fetsch et al. 2010 for a review [19]). Referred to our experiment, the prediction ${\hat{\sigma }}_{SCC+OT}^{2}$ of the ‘multi-cue’ condition is obtained by combining the ‘single-cue’ conditions σ_SCC_ and σ_OT_ according to:

$${\hat{\sigma }}_{SCC+OT}^{2}=\frac{\sigma_{SCC}^{2}*\sigma_{OT}^{2}}{\sigma_{SCC}^{2}+\sigma_{OT}^{2}}$$ Eq. 2

To investigate whether the optimal observer theory could explain our data, we therefore compared the predicted ‘multi-cue’ result ${\hat{\sigma }}_{SCC+OT}^{2}$ to the existent estimated values σ_SCC+OT_ obtained from our subjects.

#### Statistical analysis

Wilcoxon rank-sum test was used to compare σ and λ estimated in the three conditions by PD and healthy subjects, as the Lilliefors test rejected the hypothesis of normal parameter distribution. A comparison between patients and healthy subjects was also conducted within each condition separately. Wilcoxon signed rank test was instead used to test the hypothesis that the differences between the values of sigma obtained from the ‘multi-cue’ condition (σ_SCC+OT_) and those estimated according to the optimal observe theory (${\hat{\sigma }}_{SCC+OT}^{2})$ had a zero median. Bonferroni correction was used to adjust p-values for multiple comparisons, both within group, when testing differences between the three conditions, and when the two groups where compared for each condition. The quality of the fit of the cumulative Gaussian function was evaluated using R^2^.

## Results

Using a two-alternative forced-choice discrimination task (2AFC) [17] we investigated the ability of 11 patients affected by Parkinson’s disease (PD) and 19 age-matched healthy subjects to discriminate two consecutive forward tilts along the pitch plane. In the following we will first quantitatively describe the results obtained from PD patients and healthy subjects. For each population, we will subsequently investigate their performance within the context of optimal observer theory for ‘multiple cue’ integration. This latter analysis aims at exploring whether semicircular (SCC) or otolith (OT) cues are sufficient to accurately discriminate pitch tilts or whether perception would improve by a combination of both cues.

Tilt perception performance in one healthy subject and one PD patient (patient index ID5) are plotted for the three conditions in Fig 2. Data points represent the percentage of times the subject perceived the comparison stimulus as larger than the reference stimulus plotted as a function of the difference of vestibular sensory cues available for the decision. Such differences are defined by the different activations of the vestibular sensors between the two stimuli in each trial. Therefore, in the ‘multi-cue’ condition, they result from both the OTs and SCCs since both sensors are activated according to the magnitude of the ‘pure’ forward tilts. In condition ‘single-SCC-cue’ and ‘single-OT-cue’, instead, the differences result from only SCC activation and only from OT activation, respectively. If the subject behaved ideally, i.e. made no error, the curve would pass through 0% at—1 and—2 and at 100% at + 1 and + 2 of the abscissa. Or in other words, at—1 and—2 the comparison stimulus would correctly never be perceived as a larger tilt than the reference stimulus, as the vestibular cues would be minor in the comparison stimulus. Conversely, at + 1 and + 2 the comparison stimulus would always be perceived correctly as the larger tilt since the comparison stimulus would always activate the vestibular cues more than in the reference stimulus. As can be seen in in Fig 2, even the healthy subject always shows a certain grade of uncertainty, most likely, due to the inherent noise of sensory signals. Uncertainty, however, was greater in the PD patient. Responses of each single patient and healthy subject in each comparison are reported in S2 Table.

Comparison between the three conditions over all subjects revealed a similar behavior for both populations (Fig 3), except for the pronounced asymmetry of the psychometric curve (see data points connected by gray lines; note that data points and curves represent tilt performance over all trials) observed in healthy subjects in the ‘single-SCC-cue’ condition. This latter is somehow unexpected since the difference, in terms of angular rotation, between the comparison and reference stimulus is equal in the conditions where the comparison stimulus is 2 deg (and 1 deg) smaller and 2 deg (and 1 deg) larger than the reference stimulus. The differences sensed in these two conditions should therefore ideally be equivalent. This peculiarity will be discussed in more detail later on.

To statistically compare the degree of certainty of subjects for each condition, i.e. to evaluate whether the combination of vestibular cues resulted in an improvement of perceptual performance as graphically suggested (see Fig 2), we fitted a cumulative Gaussian function to the psychometric curves (see dashed curves in Fig 3; quality of fit in healthy subjects: median R^2^ 0.96 [0.16] (median [MAD—median of absolute deviations]); in patients: median R^2^ 0.88 [0.27]). The variance of the Gaussian function (see methods and Eq. 1 for details on variance and its relation to certainty), in healthy subjects was significantly smaller in the ‘multi-cue’ condition (σ_SCC+OT_ = 1.09 [0.21]) compared to the ‘single-SCC-cue’ condition (σ_SCC_ = 1.51 [0.52]; Wilcoxon-rank sum test p = 0.005) and the ‘single-OT-cue’ condition (σ_OT_ = 1.50 [0.60]; Wilcoxon-rank sum test p = 0.01), i.e. tilt perception was better when both, SCC and OT signals were present. In PD patients, σ_SCC+OT_ (1.48 [0.26]) was not different from *σ*_*SCC*_ (2.63 [0.82]) and *σ*_*OT*_ (1.90 [0.97]).

When comparing healthy subjects and PD patients, significant differences were found in the ‘multi-cue’ condition (Wilcoxon-rank sum test p = 0.02) with healthy subjects discriminating tilts better than patients (i.e. σ_SCC+OT_ was smaller in controls than in PD). No difference instead was observed for both single-cue conditions.

According to the optimal observer theory, a prediction ${\hat{\sigma }}_{SCC+OT}^{2}$ of the ‘multi-cue’ condition can be derived combining the results obtained by the parameters σ_SCC_ and σ_OT_ of the single-cue conditions (see methods). Assuming our subjects performance is close to the ideal defined by the optimal observer theory, we would expect σ_SCC+OT_ obtained from the ‘multi-cue’ condition to be close to equal to the predicted ${\hat{\sigma }}_{SCC+OT}^{2}$.

The more the observed values differ from the predicted ones, on the other hand, the less the probabilistic model would explain our data, i.e. the less vestibular cue integration would improve tilt perception performance in our experiment in a Bayesian sense (see also Fetsch et al. 2010 [19]). Values of the predicted ‘multi-cue’ condition were higher than the ones obtained from the experiment in 7 healthy subjects and lower in the remaining 12 subjects. Since certainty is lower the higher σ (in our case σ_SCC+OT_)_,_ our results indicate that 12 subjects performed worse in tilt discrimination when both SCC and OT cues were available than what would be expected from optimal cue integration (predicted optimal cue integration ${\hat{\sigma }}_{SCC+OT}^{2}$ value < as observed σ_SCC+OT_ value). The median difference between the predicted ${\hat{\sigma }}_{SCC+OT}^{2}$ and the estimated σ_SCC+OT_ value over all subjects (-0.11 [0.25]), however, was not significantly different from zero, suggesting that healthy subjects in general could efficiently combine SCC and OT information to improve tilt perception. In PD, nine of eleven patients evidenced a lower predicted ‘multi-cue’ condition and overall patients median difference between the predicted ${\hat{\sigma }}_{SCC+OT}^{2}$ and the estimated σ_SCC+OT_ value (-0.18 [0.12]) was significant lower than zero (Wilcoxon signed rank test p = 0.04). The capacity of combining SCC and OT cues by matters of optimal observer theory to improve tilt perception thus seems impaired in PD patients.

From the psychometric curve we also analyzed the parameter λ, which provides an estimate of stimulus-independent errors, such as inattention or fatigue but also of somatosensory cues, and, if not considered, could introduce significant biases in estimation of σ [18]. λ for the conditions ‘multi-cue’, ‘single-SCC-cue’ and ‘single-OT-cue’ were 0.0 [0.02], 0.22 [0.08], and 0.12 [0.06] in healthy subjects and 0.0 [0.04], 0.11 [0.11] and 0.09 [0.08] in patients, respectively. λ was significantly larger in both single-cue conditions compared to the ‘multi-cue’ condition for healthy subjects (Wilcoxon-rank sum test p<0.01), while no difference was found in patients.

## Discussion

We investigated the ability to perceive forward tilt movements in patients affected by Parkinson’s disease (PD) and in healthy aged-matched subjects. We hypothesized that an important contribution to balance dysfunction in PD patients may originate from the erroneous perception of tilt movements. The perceptual deficit thereby would stem predominantly from impaired vestibular signal processing, since the latter is the dominant component responsible for balance control during dynamic situations. By exposing patients to combinations of tilt and translational movements which stimulated either, semicircular (SCC) and otolith (OT) cues independently or simultaneously, we furthermore investigated whether patients perceptual performance improved when both sensory cues were present, i.e. in conditions of enhanced sensory information availability.

We found that PD patients perceived forward tilts less accurate than healthy subjects. Vestibular perception thus seems impaired in PD patients. Specifically tilt perception did not improve as much as in healthy controls in conditions where both SCC and OT information was available compared to conditions where only SCC or OT cues were activated. As the performance in the latter single-cue conditions was similar in patients and healthy subjects, we infer that patients’ misperception most likely was due to an integration failure of the vestibular system rather than resulting from a deficiency of the peripheral vestibular system at the level of SCC and / or OT.

Presuming that a perceptual integration failure in PD patients predominantly arises from abnormal integration of vestibular cues, the question rises how this is linked to the parkinsonian state. The pathophysiological hallmark of parkinsonism is the progressive loss of dopaminergic neurons within the basal ganglia, specifically the nigrostriatal system, although other (serotonergic, cholinergic, noradrenergic) subcortical and cortical brain areas are also involved. Neurophysiological studies have long established that the basal ganglia are a key region for sensory-motor integration (see [20] for a recent review). Electrophysiological recordings, tracer injections and functional imaging, for example, have shown that projections from the somatosensory cortex are convergent in the striatum [21] and that the striatal sensory system is organized in a somatotopic fashion similar to the motor system and localized in topographical proximity to the motor areas, thus facilitating sensory-motor interaction (see [22] for a recent review). Cell activity of the striatum, furthermore, depends on whether sensory information is linked to a motor action. Some cells, specifically, are silent for a given sensory event but active when the same sensory event functions as a cue for a movement, strengthening the sensory-motor interplay [23]. Similarly, a number of imaging studies in humans have evidenced multisensory integration of auditory and visual cues within the basal ganglia [24].

Beside the basal ganglia, there is increasing evidence that brainstem-cerebellar structures also substantially contribute to the mechanisms underlying sensory-motor integration. With focus on the vestibular system, specifically, involvement of the dentate and pedunculopontine nuclei and of vermal-nodular cerebellar structures is of particular interest as they are responsive to vestibular stimulation and involved in postural maintenance [25–28]. The presence of dopamine receptors in these structures and its neuronal degeneration and abnormal dopaminergic processing in Parkinson’s disease has been shown (see Wu and Hallett 2013 for a recent review [29]). Changes of cerebellar metabolism as evidenced by PET in Parkinson patients after treadmill walking, moreover, has been reported by Hanakawa et al., 1999 [30]. The authors specifically speculated that the cerebellar hemispheric underactivation and vermal overactivation found in the study was possibly related to an alteration of gravity perception and the subsequent loss of the ability to shift the center of gravity during locomotion. Similarly, changes of blood flow in subcortical and cortical areas involved in balance control after deep brain stimulation of the pedunculopontine nucleus in patients with Parkinson’s disease has been found, and, from a clinical point of view, pedunculopontine nucleus deep-brain stimulation was effective in preventing falls in patients with advanced Parkinson’s disease [31,32].

The main finding of this study might, at first glance, appear somehow contradictory. One in fact could question why our PD patients performed worse than healthy subjects in the ‘multi-cue’ condition where the brain processes consistent vestibular signal changes, i.e. in a ‘natural’ condition, while they were as good as controls in the single-cue conditions, i.e. in more vestibular ‘demanding’, or in other words in ‘artificial’, conditions. The ‘natural’ condition, however, can only be considered ‘simpler’ assuming a normal system functioning, i.e. in a condition where subjects are able to perform SCC and OT cue fusion automatically or in other words ‘near’ optimal. Our results, however, demonstrate that central vestibular integration in fact is impaired in our patients implying that the ‘natural’ task is presumably more demanding in patients compared to healthy controls. Conversely, we propose that the ‘artificial’ condition is similarly challenging and less well compensated in patients and healthy controls, i.e. masking the deficits of PD patients evidence in postural control.

### Discrimination task asymmetries and contribution of other sensors

Besides the observed differences in the parameter σ between and within the two groups, we also found large values of stimulus-independent error rate λ in the single-cue conditions in healthy subjects, i.e. when an additional translation was added to the tilt movement in order to isolate SCC and OT responses. Although our main finding evidences how of the vestibular system processing contribute to the perceptual integration failure, it is important to note, that areas sensitive to vestibular signals generally also display multisensory properties, i.e. are also activated by somatosensory and / or visual stimuli. Convergence of these signals occurs as early as within the brainstem and cerebellum immediately at the first synapse (see Angelaki and Cullen 2008 for a recent review [33]). This suggests that the basal ganglia and brainstem-cerebellar structures, specifically, are not just relay stations of vestibular information but rather have a role in representing aspects of body position and orientation resulting from the integration of different sensory components. It, moreover, highlights the difficulty of determining the specific contribution of vestibular cues to postural control in the parkinsonian state. In fact, even though we attempted to minimize additional sensory activation by applying passive motion stimuli in darkness, our results show that we were not completely successful in eliminating proprioceptive cues. The large values of stimulus-independent error rate λ support the notion of residual proprioceptive input influencing the participants’ estimate of tilt angle and correspond to the pronounced asymmetry of the psychometric curve observed in healthy subjects in the single-SCC-cue condition. If tilt perception relied exclusively on vestibular information, we would expect a symmetric curve as the difference of vestibular cues activated by the smaller and larger comparison stimuli with respect to the reference stimulus were equal (see Fig 1). The alternative hypothesis of a distorted curve resulting from differences of perceptual thresholds for small and large tilt movements, i.e. a higher sensitivity of tilt perception for larger than smaller tilt angles, appears unlikely since we then would have expected to find a similar asymmetry also for the ‘multi-cue’ condition.

### Study limitations

While our data suggest that central vestibular processing in Parkinson’s disease is impaired contributing to balance dysfunction, some study limitations deserve further attention. Patients were left on regular medication and tested in their ON-condition. Although it is still unclear how and to what extent the dopamine system interacts with the vestibular system, in animals, dopamine has been shown to exert modulatory effects on diverse neurotransmitters within the vestibular labyrinth and the vestibular nuclei (see [34] for a recent review). The expression of dopamine receptors within the striatal system, moreover, is altered with vestibular loss. Dopamine receptors in the striatum, for example, are upregulated in vestibular hemilabyrinthectomized rats [35]. Locomotion in vestibular deficient rodents is also characteristically changed to a hyperactive and cyclic behavior, patterns related to the dopaminergic system [36] and, in line, this abnormal pattern is partially restored by drugs inhibiting the striatal dopaminergic function [37]. In humans, interestingly, acceleration of vestibular compensation after unilateral vestibular loss, i.e. a decrease of asymmetry of the vestibulo-ocular reflex determined by sinusoidal chair rotations, has been reported in patients treated with L-sulpirine, a dopamine antagonist [38]. While it is important to emphasize that the study was primarily designed to investigate the role of L-sulpirine in symptomatic control of acute vertigo spells and not to investigate vestibular-reflex behavior, these findings are accordant with another recent study reporting the worsening of proprioceptive kinesthetic processing in patients tested under regular (ON-state) vs. discontinued (OFF-state) dopaminergic medication [39]. We thus cannot exclude that dopaminergic medication influenced vestibular information processing and future studies should investigate this issue.

Another possible limiting factor consists in the heterogeneity of our patients’ disease stages (see S1 Table). It is supposable, that vestibular function and / or the ability to centrally integrate information were not affected in the same way in early or advanced stages and further studies with larger populations should also consider this topic.

Tilt perception was investigated in seated subjects. While the sitting position allowed us to safely expose subjects to dynamic perturbations, it limits the informative value on balance, and in particular on postural control. The sitting position, moreover, could have negatively impacted our effort to minimize proprioceptive cues. As discussed above, in fact, our findings suggest that we were probably not able to completely eliminate proprioceptive cues, as this is the most likely explanation for the pronounced asymmetry of psychometric curves observed in our healthy subjects in the single-SCC-cue condition. Residual proprioceptive information, thus, could have influenced our findings. Due to the dynamic stimuli chosen in this study, however, we still feel confident in assuming that the vestibular system played a major role in tilt perception.

### Conclusions

In conclusion, we have shown that vestibular signal processing is impaired in patients affected by Parkinson’s disease. Rather than being a deficit in primary vestibular feedback, it most likely results from a failure of higher order sensory integration centers, with the basal ganglia possibly constituting a core region of this integrative process.

## Supporting Information

S1 Table Patients’ characteristics. (DOCX)

S2 Table Healthy subjects’ and Patients’ responses. The table list the percentage of time the comparison stimulus was judged as larger tilt than the reference stimulus in all the conditions tested by each single patient and single subject.
(DOCX)
