# Supplementary material for: Impaired Tilt Perception in Parkinson’s Disease: A Central Vestibular Integration Failure
Source: PLoS One. 2015 Apr 15;10(4):e0124253. doi: 10.1371/journal.pone.0124253 (PMC4398395; doi:10.1371/journal.pone.0124253)
Supplement: S1 Table — (DOCX) [file pone.0124253.s001.docx]

S1_Table 1. Patients’ characteristics

**Disease duration (y) Hoehn-Yahr Stage UPDRS motor score Mini Mental State anti-Parkinsonian medication (maximal dose in mg)**

**Patient ID/Age/Sex**

1/56/M 11 4 39 26 Levodopa/Benserazide (1000 / 250)

Ropirinole (12)

2/80/M 15 2 39 27 Levodopa/Carbidopa (600/150)

Ropirinole (3)

3/67/M 10 2 21 n.p. Levodopa/Carbidopa (1000/250)

Selegiline (5)

4/69/M 7 3 21 29 Levodopa/Benserazide (600/150)

5/66/M 8 2 12 30 Levodopa/Benserazide (700/150)

Pramipexole (4.5)

6/68/M 6 2 20 29 Levodopa/Carbidopa/Entacapone (750/187.5/1000)

Pramipexole (3)

7/69/M 5 3 20 26 Levodopa/Carbidopa/Entacapone (600/150/1600)

Ropirinole (12)

Amantadine (200)

8/70/F 2 1 15 27 Levodopa/Benserazide (600/150)

Pramipexole (4.5)

9/67/M 5 2 25 28 Levodopa/Carbidopa/Entacapone (400/100/800)

Pramipexole (1)

10/64/M 4 1 15 28 Levodopa/Benserazide (300/75)

Pramipexole (0.75)

11/43/F 2 1 15 30 Levodopa/Carbidopa (600/150)

Pramipexole (4.5)

Legend: F = female; M = male; n.p. = not performed;
