# Supplementary material for: Impaired Tilt Perception in Parkinson’s Disease: A Central Vestibular Integration Failure
Source: PLoS One. 2015 Apr 15;10(4):e0124253. doi: 10.1371/journal.pone.0124253 (PMC4398395; doi:10.1371/journal.pone.0124253)
Supplement: S2 Table — The table list the percentage of time the comparison stimulus was judged as larger tilt than the reference stimulus in all the conditions tested by each single patient and single subject. (DOCX) [file pone.0124253.s002.docx]

S2_Table 2. Healthy subjects’ and Patients’ responses. The table list the percentage of time the comparison stimulus was judged as larger tilt than the reference stimulus in all the conditions tested by each single patient and single subject.

|  | **Multi-cue condition** | | | | **Single-SCC-cue condition** | | | | **Single-OT-cue condition** | | | |
| --- | --- | --- | --- | --- | --- | --- | --- | --- | --- | --- | --- | --- |
|  | **-2** | **-1** | **1** | **2** | **-2** | **-1** | **1** | **2** | **-2** | **-1** | **1** | **2** |
| **Patient ID** |  |  |  |  |  |  |  |  |  |  |  |  |
| **1** | 0 | 0 | 94 | 94 | 56 | 56 | 75 | 100 | 19 | 19 | 94 | 100 |
| **2** | 0 | 17 | 92 | 83 | 8 | 75 | 67 | 92 | 42 | 25 | 75 | 100 |
| **3** | 0 | 21 | 71 | 88 | 33 | 38 | 71 | 79 | 33 | 21 | 88 | 83 |
| **4** | 29 | 24 | 82 | 70 | 32 | 62 | 52 | 90 | 33 | 43 | 55 | 65 |
| **5** | 6 | 13 | 75 | 94 | 25 | 31 | 50 | 94 | 13 | 31 | 75 | 69 |
| **6** | 10 | 15 | 65 | 75 | 45 | 45 | 35 | 65 | 20 | 35 | 65 | 90 |
| **7** | 75 | 25 | 88 | 50 | 25 | 50 | 63 | 38 | 38 | 63 | 50 | 63 |
| **8** | 38 | 50 | 50 | 56 | 31 | 19 | 44 | 63 | 69 | 25 | 50 | 56 |
| **9** | 0 | 13 | 38 | 100 | 38 | 38 | 88 | 88 | 63 | 25 | 75 | 100 |
| **10** | 0 | 38 | 75 | 88 | 13 | 13 | 75 | 88 | 25 | 25 | 71 | 63 |
| **11** | 13 | 19 | 69 | 94 | 69 | 44 | 75 | 88 | 19 | 19 | 81 | 100 |
| **Healthy subject ID** |  |  |  |  |  |  |  |  |  |  |  |  |
| **1** | 0 | 29 | 88 | 100 | 13 | 50 | 75 | 88 | 53 | 56 | 78 | 71 |
| **2** | 13 | 38 | 56 | 81 | 6 | 19 | 81 | 94 | 19 | 63 | 63 | 81 |
| **3** | 10 | 25 | 80 | 90 | 55 | 45 | 50 | 85 | 25 | 50 | 70 | 80 |
| **4** | 8 | 25 | 79 | 88 | 71 | 67 | 63 | 92 | 13 | 17 | 79 | 92 |
| **5** | 0 | 17 | 83 | 83 | 33 | 8 | 75 | 100 | 58 | 42 | 75 | 83 |
| **6** | 0 | 4 | 88 | 96 | 21 | 17 | 63 | 100 | 17 | 46 | 63 | 83 |
| **7** | 8 | 17 | 100 | 100 | 33 | 25 | 75 | 100 | 42 | 25 | 67 | 83 |
| **8** | 17 | 8 | 83 | 92 | 42 | 17 | 83 | 100 | 17 | 25 | 50 | 83 |
| **9** | 0 | 19 | 69 | 100 | 81 | 75 | 56 | 100 | 19 | 13 | 100 | 100 |
| **10** | 0 | 8 | 75 | 92 | 67 | 42 | 58 | 100 | 25 | 8 | 92 | 92 |
| **11** | 0 | 5 | 80 | 95 | 55 | 30 | 80 | 95 | 15 | 25 | 95 | 95 |
| **12** | 0 | 6 | 81 | 100 | 44 | 13 | 81 | 100 | 13 | 13 | 81 | 94 |
| **13** | 0 | 25 | 92 | 92 | 42 | 42 | 75 | 91 | 8 | 25 | 75 | 100 |
| **14** | 17 | 35 | 75 | 71 | 42 | 33 | 67 | 87 | 71 | 70 | 57 | 79 |
| **15** | 4 | 4 | 75 | 92 | 46 | 54 | 58 | 71 | 33 | 25 | 71 | 96 |
| **16** | 5 | 23 | 86 | 86 | 55 | 48 | 68 | 86 | 23 | 19 | 77 | 86 |
| **17** | 4 | 9 | 83 | 96 | 8 | 29 | 75 | 96 | 29 | 29 | 71 | 96 |
| **18** | 5 | 10 | 80 | 90 | 60 | 35 | 75 | 100 | 30 | 35 | 85 | 90 |
| **19** | 0 | 0 | 90 | 100 | 40 | 20 | 90 | 90 | 10 | 10 | 80 | 100 |
